# Marketed Marine Natural Products in the Pharmaceutical and Cosmeceutical Industries: Tips for Success

**DOI:** 10.3390/md12021066

**Published:** 2014-02-17

**Authors:** Ana Martins, Helena Vieira, Helena Gaspar, Susana Santos

**Affiliations:** 1 BIOALVO, S.A., Tec Labs Centro de Inovação, Campus da FCUL, Campo Grande, Lisboa 1749-016, Portugal; E-Mails: ana.martins@bioalvo.com (A.M.); hmvieira@fc.ul.pt (H.V.); 2 Center for Biodiversity, Functional & Integrative Genomics (BioFIG) and Departamento de Estatística e Investigação Operacional (DEIO), Faculdade de Ciências, Universidade de Lisboa, Campo Grande, Lisboa 1749-016, Portugal; 3 Centro de Química e Bioquímica (CQB) and Departamento de Química e Bioquímica (DQB), Faculdade de Ciências, Universidade de Lisboa, Campo Grande, Lisboa 1749-016, Portugal; E-Mail: hmgaspar@fc.ul.pt

**Keywords:** marine natural products, pharmaceuticals, cosmeceuticals, biodiscovery, challenges, success, market

## Abstract

The marine environment harbors a number of macro and micro organisms that have developed unique metabolic abilities to ensure their survival in diverse and hostile habitats, resulting in the biosynthesis of an array of secondary metabolites with specific activities. Several of these metabolites are high-value commercial products for the pharmaceutical and cosmeceutical industries. The aim of this review is to outline the paths of marine natural products discovery and development, with a special focus on the compounds that successfully reached the market and particularly looking at the approaches tackled by the pharmaceutical and cosmetic companies that succeeded in marketing those products. The main challenges faced during marine bioactives discovery and development programs were analyzed and grouped in three categories: biodiversity (accessibility to marine resources and efficient screening), supply and technical (sustainable production of the bioactives and knowledge of the mechanism of action) and market (processes, costs, partnerships and marketing). Tips to surpass these challenges are given in order to improve the market entry success rates of highly promising marine bioactives in the current pipelines, highlighting what can be learned from the successful and unsuccessful stories that can be applied to novel and/or ongoing marine natural products discovery and development programs.

## 1. Introduction

Natural products (NP) are usually small molecules, with a molecular weight below 3000 Da, which are produced by a biological source such as plants, animals and microorganisms, but which occurrence may be limited to a particular taxonomic family, genus, species or even organism [1]. They are often called secondary metabolites because, predominantly, they are not biosynthesized by the general metabolic pathways and have no primary function directly involved in the normal growth, development or reproduction of an organism. They are generally used by organisms to control ecological relationships that involve defense against predation, competition for space and food, interspecies communication for the purposes of mating, hunting or quorum signaling, among other functions. NP have long been a traditional source of medicines, and are still nowadays considered the most successful supply of potential drug leads with more than 1 million new chemical entities discovered so far [2,3]. Historical examples of early identified natural compounds are undoubtedly the isolation of morphine from *Papaver somniferum* poppies, first reported in 1803, and the discovery in 1929 by Flemming of the first antibiotic penicillin from the fungus *Penicillium* *notatum* [3]. Since then, numerous other NP have been isolated and identified with 60% of the drugs currently on the market being of natural origin [4]. These compounds are known to present several advantageous as compared with non-natural compounds such as high chemical diversity, biochemical specificity, binding efficiency and propensity to interact with biological targets, which make them favorable lead structures.

Suitable natural sources for the discovery of new potentially bioactive molecules are numerous, but marine environment, harboring a vast variety of organisms differing in their physiology and adaptation capacity, is becoming a top spot for the identification of new drug leads. From the over 33 animal phyla described to date, 32 are represented in the aquatic environment, with 15 being exclusively marine [5]. Despite the fact that oceans cover more than 70% of the earth’s surface, the exploration of marine ecosystems has only began in the mid 1970’s, with the emergence of modern snorkeling, the introduction of scuba in 1970 and later, around 1990, with the use of remotely operated vehicles (ROVs) [2]. Due to technical limitations, exploitation of marine organisms started with the collection of large creatures such as red algae, sponges and soft corals, which were shown to produce a large variety of compounds with quite unique chemical structures [6]. Invertebrates alone comprise approximately 60% of all marine animals and were described as the source of more than 11,000 new NP since 1990 [7,8,9]. With the continuous exploitation of the marine environment, attention turned to microorganisms such as marine cyanobacteria, marine fungi, and several other groups of marine bacteria due to their biological and habitat diversity, which resulted in the ability to produce metabolites with unmatched structures [10]. Microorganisms constitute nowadays a prolific source of structurally diverse bioactive metabolites and have yielded some of the most important active ingredients known today [11]. Recently it was even realized that many compounds previously isolated from marine macroorganisms, such as sponges and tunicates, are in fact, metabolic products of associated microorganisms [12,13].

Due to their broad panel of bioactivities such as anti-tumor, anti-microtubule, anti-proliferative, photoprotective, antibiotic and anti-infective [14,15,16,17,18,19], marine natural products (MNP) are exceptionally interesting high-value ingredients for applications in the pharmaceutical industry and more and more companies are investing in this field. Following the same trend, cosmetics industry is progressively turning to the sea in the search for new ingredients. Traditionally, in the field of cosmetic industry cosmetics were defined as articles to be applied to human body for cleansing, beautifying, promoting attractiveness, or altering the appearance without affecting body structure or functions [20]. However, more recently, the cosmetic industry introduced a special class of products, the cosmeceuticals, as a combination of cosmetics and pharmaceuticals, as bioactive ingredients are now combined with creams, lotions and ointments [21]. Interestingly, an increasing number of suppliers of the cosmetic industry are being pushed to include extracts made from costal plants, seaweeds, algae and sea minerals into cosmeceutical ingredients. These extracts contain vitamins and minerals and they show ultraviolet and anti-oxidant protection and general anti-aging benefits [22,23,24,25]. In fact, activities such as antioxidant, anti-wrinkle, anti-tyrosinase and anti-acne are among the most usual activities of marine cosmetic ingredients for skin health [21,26]. Hence, an entire new paradigm of beauty care, combining cosmetics and pharmaceuticals properties into novel products with biologically active ingredients, will be the hallmark of the next decades.

The aim of this review is to outline the role of MNP in pharmaceutical and cosmeceutical industries, to identify the main bottlenecks found during the process of discovery and development, and to give an overview over the compounds that entered successfully in those markets. Tips for success will also be given so that more MNP can reach the market.

## 2. Marine Environment as an Unexploited Source for Bioactives Discovery

The discovery and identification of the two nucleosides spongothymidine and spongouridine in the early 1950s from the Caribbean marine sponge *Cryptotethia crypta* paved the way of MNP as promising new chemical entities of potential therapeutic value [27]. Since then, several other therapeutic agents have been obtained through isolation from natural sources, by chemical synthesis or a combination of both.

In the most traditional process for bioactive discovery, a natural product is firstly extracted from the source, screened against a specific target, isolated by a bioassay-guided isolation procedure, fractionated and purified, yielding essentially a single biological active compound. Despite its widespread use, this traditional method of natural product bioactive discovery is slow, labor intensive, barely efficient and provides no guarantee of success. Nowadays, NP discovery is on high demand for rapid screening, hit identification and hit-to-lead faster development processes, being mandatory to explore new approaches in order to compete successfully with other alternative drug discovery methods. In fact, rational drug design involving high throughput screening (HTS) technology in combination with combinatorial chemistry (CC) have reduced in the past decades the interest on NP discovery [28]. The two techniques together make the screening of synthetic compounds faster and cheaper as compared to traditional natural product discovery [29]. Nevertheless, such rapid synthetic techniques have not led to successful development of bioactives. For instance, early CC libraries were composed of compounds with poor solubility, low purity and short chemical diversity and hence only a few useful hits were identified [28,29]. So, some companies, driven by achievements on total or hemi-synthesis of NP [29], development of synthetic analogues or design of synthesizable pharmacophores of reduced complexity, brought back natural product discovery programs [30,31].

The development of refined analytical and spectroscopic methods, particularly high resolution nuclear magnetic resonance (NMR) and mass spectrometry (MS) has also contributed tremendously to put NP, and in particular MNP, back on the track of drug discovery [32]. In fact, the many advances in spectroscopic methods, allowed the *de novo* structure determination of new chemical entities (NCEs) in very small concentrations [33] even in complex mixtures as crude extracts.

An additional fact that helped to put MNP back on the agenda of drug discovery programs was the recent development of techniques that allow the access to a vast community of microbial sources unattainable until recently. Culture broths of new microorganisms are now very easy to obtain, through small scale, high-throughput cultivation methods that use nutrient deficient media, specific nutrients and long cultivation times [34]. In addition, as more than 99% of the microorganisms is not readily cultivable, culture independent methods based on the total DNA of the bacterial community, can now detect a considerable fraction of the uncultivable organisms (in addition to those that can be cultured) enabling the access to these previously unreachable natural product resources. Metagenomics, in particular, enables direct access to the genomes of whole environmental uncultivable microorganisms by total environmental DNA extraction [35] and it has already proven to be a good alternative for exploiting uncultivable microorganisms for natural product discovery [36]. Ultimately, genome mining, which consists in the analyses of genome sequences for the identification of genes encoding proteins, is an additional recent approach which has allowed the discovery of numerous novel NP and also revealed gene clusters and novel pathways for the biosynthesis of several known natural compounds [37]. The biosynthesis of polyketides, for example, has attracted most attention, since many commercial antibiotics derive from this pathway. Genome mining is of high importance on NP discovery as it opens up the possibility of expressing the gene clusters in a heterologous organism, obtaining the desired secondary metabolites [38]. Furthermore, it has also allowed the development of combinatorial biosynthesis, which involves the genetic manipulation of the gene cluster involved in NP biosynthesis to obtain new molecules that would be difficult to synthesize using other methods [39].

All the progressive improvements in the past 50 years of exploration of the marine environment, pointed out earlier, have resulted in the isolation of approximately 20,000 structurally unique bioactive MNP [40]. Just in 2012, 1241 new compounds were reported which clearly identifies the marine environment as a rich source of bioactive molecules [8]. Nevertheless, despite this enormous number of structurally unique bioactive MNP, to the date the global marine pharmaceutical pipeline includes only eight approved drugs, twelve NP (or derivatives thereof) in different clinical phases and a large number of marine chemicals in the preclinical phase [6,41,42]. The global market for marine-derived drugs is forecasted to reach $8.6 B by 2016 [43].

In alignment with the pharmaceutical industry, the cosmeceutical industry is increasingly turning to the sea in the search for new molecules. In fact, MNP are on high demand for skin care, as they are scientifically advanced and environmentally friendly, besides offering a variety of benefits. Seven types of cosmeceutical ingredients derived from marine resources have been identified so far, and are currently commercialized [21]. Driven by an aging population looking for innovative anti-aging solutions, the global cosmeceuticals in the US has reached $13.1 B in 2011 and is projected to grow almost twice the average rate of cosmetics sector [44].

In conclusion, MNP constitute a strategic research area with enormous economic and social revenues, where scientists all over the world are interested. According to EuroOcean there are over 590 European marine projects funded by both FP6 and FP7 actions [45], which shows the engagement of academia and industry in bringing more marine bioactives into the market.

## 3. Challenges Faced during Marine Natural Products Development

For the purposes of this review, the major challenges faced by discovery and development programs of new bioactives from marine biological resources were grouped in three categories: biodiversity, supply and technical, and market. In the topic “biodiversity”, a focus is addressed on challenges associated with secure access to marine resources, with the correct identification of the biological material and with the efficient screening of samples and compounds. The topic “supply and technical” covers the challenges associated with the actual process of isolation and sustainable production of the pure bioactive, and the understanding of its mechanism of action towards the desired target). Finally, in the topic “market challenges” considerations are made on the process and the costs of developing a NP bioactive [46].

### 3.1. Biodiversity Challenges

It is widely accepted that we know more about the moon than we actually know about the sea. The access to the ocean and to the deepest of its spots remains very difficult and further robotic and engineering technology is needed to fully evaluate the oceans available biodiversity. Because traditional medicinal knowledge associated with marine organisms is almost nonexistent, the search for biologically active compounds from marine sources has occurred via a random selection of organisms. Three different sampling approaches are commonly used: (i) exploring unexploited taxonomical groups or geographical sources, in order to maximize the chances of finding new molecules; (ii) exploring new taxa and/or regions of confirmed chemical diversity; or (iii) combining both of these strategies [47,48]. In either case, substantial knowledge of bio- and chemo-diversity is required.

Marine natural product studies were formerly conducted, randomly, in shallow coastal waters, which left aside an enormous community of very promising organisms which lived in untapped but unreachable environments [49], e.g., hydrothermal vents and sea mounts. Sampling in difficult access spots, deeper than 30 m, is not possible by scuba diving and is usually performed by trawling. This technique besides being unselective, often damages the samples and the benthic ecosystems, and hides valuable information about the environment of the organism [50]. The ideal sampling of marine organisms in deep waters is by the use of sophisticated or ingenious equipment, such as manned submersibles and, more recently, ROVs [2]. With this kind of equipment it is possible to observe the organism’s habitat, and to foresee a possible bioactivity for its metabolites. For instance, if it is observed that a sponge lays alone in the habitat, and around it there is a clean area, most probably, its metabolites inhibit the growth of competing organisms [50]. Unfortunately, these sampling facilities are very expensive and only a small number of laboratories have access to them, a drawback that is difficult to overcome, especially if we think that the majority of biological diversity is located in underdeveloped countries from the tropical and subtropical regions [51]. This is just one of the reasons why international collaboration is so important in this research field. However, the access to biodiversity on natural resources is now under the host of the Convention on Biological Diversity (CBD). Unfortunately, the different levels of Nagoya protocol implementation (which clarifies the scope of the CBD) in different regions, and the increasing difficulties to work under a still unclear regulatory framework on biodiversity access may push current industries out of the NP arena.

The lack of taxonomic knowledge for marine species, and the still large number of unidentified species and strains, is also a major blockage faced by marine natural products programs. The selection, for pharmacological purposes, of macro or microorganisms, either terrestrial or marine, must be grounded on a correct taxonomic identification and classification. An incorrect classification of a species may compromise an entire drug discovery project, not only because it is impossible to reproduce the isolation in the event of a bioactive extract and/or metabolite, but also because it can mislead the dereplication process—the process by which the bioactives are identified. Approaches to classification of marine macroorganisms (algae and invertebrates) and microorganisms (fungi and bacteria) are quite different. For the majority of marine macroorganisms taxonomic knowledge is still insufficient to enable unambiguous species classification [52]. Macroinvertebrates are especially challenging, not only due the fact that there are still many undescribed species, but also because many related species must be distinguished based on subtle morphological characteristics [53].

Following the process of target identification and validation, the next step of a drug discovery process is the development of the screening assays. A variety of screening paradigms exist to identify hit molecules [54] being HTS the most widely used in the case of NP. The success key to apply HTS methodology to NP is constructing high quality libraries. Researchers at Pfizer proposed that the output from HTS is dependent on the interrelationships between the quality of the compound library, the target and the screening process [55]. Ideally, the library itself should be composed by crude extracts, simplified extract fractions and pure compounds for a well-balanced natural product discovery program [56]. Crude extract libraries are easier and cheaper to construct, have moderate overall size and a high degree of diversity, but have major disadvantages when compared with pure compounds libraries. Crude extracts are complex mixtures of several compounds that may have synergistic interaction, a fact that accounts for the disappearance of the bioactivity in purified fractions and, ultimately, in final pure compounds [57]. On the other hand, false negative readouts may also be obtained, either because an active metabolite is present in a small percentage in the crude extract, or because of the interference of compounds such as tannins [56] that bind to other metabolites masking its activity. Due to these reasons, in a recent past this approach was discouraged in drug discovery programs [56]. Screening pre-fractionated libraries is an effective strategy to avoid these problems [3]. Depending on the method used for pre-fractionation and on the number of compounds in the original crude extract, the resulting fractions can vary widely in complexity from a mixture of multiple compounds to a single major compound of >90% purity. Pre-fractionation can eliminate several undesired compounds and facilitate hit identification.

All in all, the association of modern HTS methods and robust NP libraries, representative of a wide biodiversity, is a powerful tool to streamline cosmeceutical and therapeutic lead discovery programs.

### 3.2. Supply and Technical Challenges

Several different problems are associated with supply and technical issues. The first one is related to the variability of the organism itself. For instance, taking the example of sponges, the high frequency of their bioactive metabolites is interpreted as chemical defense against environmental stress factors such as predation, overgrowth by fouling organisms or competition for space. The highest incidence of toxic or deterrent sponge metabolites is found in habitats such as coral reefs that are characterized by intense competition and feeding pressure. Because these environmental conditions are not static, it is likely that a resupply of the same organism does not provide the same metabolite. Also, in the case of marine invertebrates another challenge is the fact the microorganisms are sometimes the actual producers of the bioactives.

Once a particular natural product has been isolated and identified as a lead compound, the issue of its sustainable supply is faced. Most of the times, the compound of interest is present only in low amounts and/or can be very difficult to isolate [17]. In the case of tissues of marine invertebrates, which present unique extraction-related problems due to their high water and salt content, this problem can be even more challenging. Whatever the use of the compound (drug, cosmetic, *etc*.), several grams to hundreds of grams are required for preclinical development, multikilogram quantities are needed for clinical phases and tons for cosmetic uses.

Mariculture (favoring by farming the growth of the organism in its natural milieu) and aquaculture (culture of the organism under artificial conditions) have been attempted in order to solve the problem of sustainable supply of macroorganisms. However, the unique and sometimes exclusive, conditions of the sea make cultivation or maintenance of the isolated samples very difficult and often impossible. For example, sponges and their microbiota are generally not suitable for cultivation, hence, the compound of interest may need to be extracted and purified from the specimens collected in the wild [47]. These constraints lead to the loss of a major portion of the available marine biodiversity and represent a major bottleneck in the sustainable supply of the desired natural compound.

This lack of sustainable supply of substances has stopped further development of several highly promising marine compounds, and attempts have been made to overcome this barrier by developing synthetic or hemisynthesic analogues, derivatives with more manageable properties, or by design of a pharmacophore of reduced complexity which can then be synthesized [30]. However, it is worth noting, that these approaches embrace themselves their own challenges. Total synthesis is by no means an easy undertaking task, and chemistry still has a very long way to go before it can make any molecule in a practical manner. NP are complex and exquisite molecules possessing, almost always, one or several stereocenters, a fact that renders their synthesis hard to achieve, since enanteo or diastereoselective synthetic or purification processes are difficult to perform. Hemisynthesis may be, in some cases, a good solution for compound’s supply. This process involves harvesting a biosynthetic intermediate from the natural source, rather than the lead itself, and converting it into the lead. This approach has two advantages. First, the intermediate may be more easily extracted in a higher yield than the final product itself. Second, it may allow the syntheses of analogues of the final product.

Additionally, the synthesis or hemisynthesis of a bioactive natural compound must be supported by a correct identification of the compound isolated from the biological source. Despite the fact that modern methodologies of structural elucidation are well advanced, errors can never be completely ruled out. In fact, there are numerous structural revisions reported in literature, even of recently elucidated NP. In average, per 5-year period, 369 NP and 135 MNP are misassigned [58]. The structural complexity of the isolated compounds and the small amount of samples, especially in the case of compounds from marine sources, can contribute to misassignments which can be divided in several categories: incorrect formula, constitution (planar connectivity), double bond configuration, absolute configuration, and one or several stereocenters assigned incorrectly [59].

To avoid “rediscovery of the known” more specialized and effective dereplication strategies need to be employed. With over 150,000 small molecules characterized from natural sources, previously known natural compounds are often re-isolated during bioassay-guided fractionation and that should be avoided [29]. Hyphenated technics such as liquid chromatography with ultraviolet detection (LC-UV), liquid chromatography-mass spectrometry (LC-MS, LC-MS/MS) or LC-NMR are valuable tools for the dereplication process, especially if used early in the prefractionation step [60]. Access to suitable databases is essential for the rapid dereplication of crude extracts in natural product research. Several commercial databases are available to implement the dereplication process, from which the most comprehensive ones are: Chemical abstracts, including NAPRALERT, Beilstein, AntiBase [61] (>40,000 natural compounds from micro-organisms and higher fungi), MarinLit (~24,000 marine compounds isolated from approximately 6000 species) [62], Chapman & Hall’s Dictionary of Natural Products (~170,000 compounds from both marine and terrestrial organisms) [63] and NAPROC-13 (^13^CNMR spectral information of over 6000 natural compounds) [64].

When using pure natural compound libraries, virtual screening is also a possibility that must be stressed out. Virtual screening can be used for browsing databases in the quest for molecules fitting either an established pharmacophore model or a three dimensional structure of a macromolecular target. The advantages of this approach over *in vitro* screening are obvious: higher capacity, no need for physically isolating the compounds, less time-consuming and expensive and theoretically, interactions of all compounds to all structurally defined targets can be calculated and predicted. Additionally early evaluation of absorption, distribution, metabolism, and excretion/toxicity in pharmacokinetics (ADMET) properties is also possible [65]. But, because virtual screening is only a predictive tool, in the case of NP it is important the integration of *in silico* screening with traditional avenues, gathering information from bioassay guided fractionation, on-line analytical activity profiling, ethnopharmacological screening, if it is the case, and chemoinformatics, in order to achieve an optimization of drug lead discovery [65].

Finally, upon the identification of a lead it is necessary to understand its mode of action against the specific target. This includes secondary testing in which molecular and cellular techniques are normally applied. This identification constitutes a major challenge but is becoming more and more compulsory in both pharmaceutical and cosmeceutical industries.

### 3.3. Market Challenges

Finally the commercial and market issues are very relevant and most of the times disregarded in the natural product development programs. Since the very early stages of the development programs, several very important questions must be addressed by the researchers or the companies: (i) what are the potential industry applications and the market need of that particular activity; (ii) what is the target price/kg of the final bioactive; (iii) what is the formulation desired and the route of administration; (iv) what is the manufacturing process and how sustainable is the supply; (v) how can the product reach the market value chain. The high number of NP hits and leads coming out of the HTS technologies has stressed out the need for a focused strategy on this field. Small and Medium Enterprises (SME’s) have a commercialization goal and, therefore, introduce very early on their discovery and development programs the issues indicated earlier. It is crucial for them to have a clearly defined strategy, otherwise the risk of failing and running out of cash fast is high. It is important to be aware that the cost of technology and manufacturing processes, sometimes with poor yields, raises the market cost per kilogram and may render these products economically unviable. This is particularly true in personal care industry where recombinant technologies are not acceptable and the profit margins are too small to introduce very expensive ingredients per pack. Recently, academia has started to be more aware of the “market issues” as it became obvious that most of the discoveries on the NP pipeline where barely reaching the market and consumers. This has been partially achieved by serious encouragement of industry-academia partnerships, both at national and transnational levels that went beyond the traditional funding by the industry of small research projects of academia. These alliances benefit both partners that work side-by-side, with a common set of goals and in a win-win collaboration system. Academia gets knowledge, publications and funding and industry gets new NP with higher probabilities of market success.

Bearing in mind all the challenges just pointed out, NP developers can thrive to find better models of development to surpass or minimize their impact.

## 4. Marketed Marine Natural Products. Examples of Success Stories

The Marine Board of the European Science Foundation has published a position paper in which it provides a roadmap for European research in Marine Biotechnology and sets out an ambitious science and policy agenda for the next decade [66]. Development of novel drugs, treatments and health and personal care products, is one of the five research areas prioritized in this document that can greatly contribute to key societal challenges.

An overview of marine drugs and cosmeceuticals that successfully reached the market is the focus of next section. Hopefully, the analysis of the issues related with their development will allow a deeper understanding of the key factors behind their success.

### 4.1. Pharmaceutical Applications

As pointed earlier, natural product screening remains one of the most useful avenues for bioactive discovery. In the past decades, studies on MNP have been focused mainly on macroorganisms, *i.e.*, sponges, corals and other marine invertebrates, although significant developments have been made in the microorganisms area. However, despite the large number of NCEs isolated from marine organisms, many of them with pronounced biological activity, the great majority does not surpass the pharmaceutical pre-clinical trials and only a very few have been marketed as pharmaceutical products.

**Figure 1:**
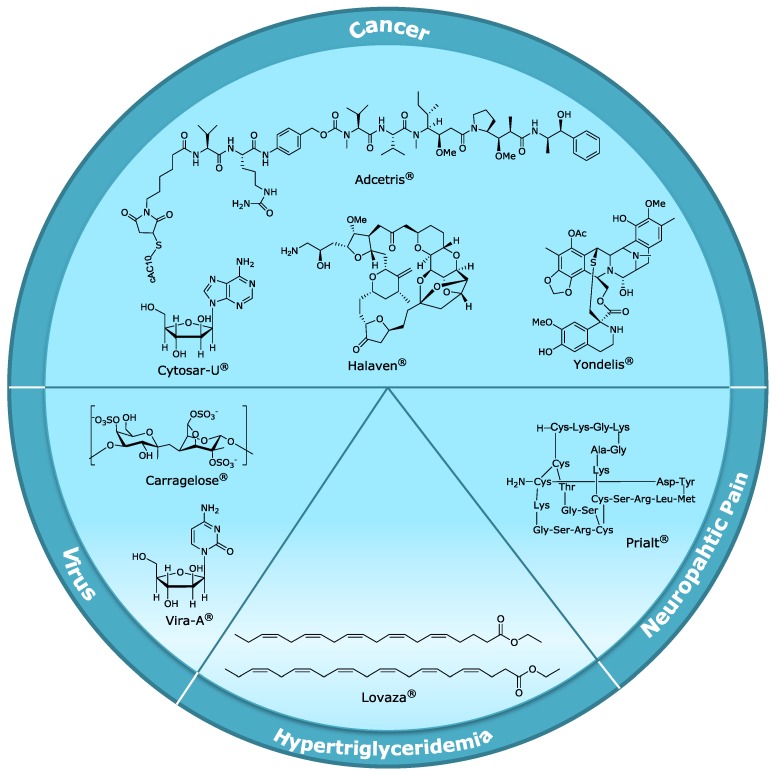
Chemical structures of marine drugs on the market divided by therapeutic area.

Besides the usual drawbacks in any drug discovery process, the industrial development of many promising MNP was hampered by additional difficulties such as sustainable source and issues related to structural complexity and scale up. Nevertheless, the global marine pharmaceutical pipeline remains very active and includes, at the moment, eight Food and Drug Admnistration (FDA) or European Medicines Agency (EMEA) approved drugs and several compounds in different phases of the clinical pipeline [67]. From the eight compounds currently on the market (Figure 1), only three (Prialt^®^, Yondelis^®^ and Carragelose^®^), became drugs without any modification of the original natural molecule, while the rest of them suffered lead optimization, in different steps of their development. Overall, from lead discovery to the entry in the market it took 20 to 30 years. Ensuring natural product supply on an industrial scale, optimization of formulation and ADMET properties were the main blockades faced by pharmaceutical companies. Optimization of NP by structural modifications, synthetic supply of unmodified natural molecule or immunoconjugation of NP was the strategy behind these successful stories. The history of the development of the market drugs will be discussed in this subsection with a focus on the approaches tackled by the pharmaceutical companies that succeeded in marketing their products.

Table 1 highlights the evolution of this pipeline from 2004 until now. This pipeline is quite dynamic with a significant and growing number of marine-derived molecules entering in clinical trials, and some others being discontinued for several causes. The main reasons for the withdrawals of development in clinical phases I–III are mainly due to lack of efficacy (43%) and drug toxicity (33%) [68]. In 2004 cytarabine, vidarabine, ziconotide and omega-3-acid ethyl esters were the only FDA/EMEA approved drugs. Just nine years later, the approved drugs in the marine drug pipeline doubled. This is a amazing improvement since during the first 30 years of MNP research, cytarabine and vidarabine were the only ones entering the market. Just three compounds in clinical trials in 2004 remain in the pipeline: pliditepsin, DMXBA (GTS-21) and bryostatin I.

#### 4.1.1. Cytosar-U^®^ and Vira-A^®^ by Bedford Laboratories (Bedford, OH, USA) and King Pharmaceuticals (Tenafly, NJ, USA), Respectively

The presence of an arabinose unit instead of ribose in the nucleosides spongouridine and spongothyminide was the pitfall that inspired the synthesis of several ara-nucleosides that steered the development of the marketed drugs cytarabine (FDA approved in 1969 for cancer) and vidarabine (FDA approved in 1976 as antiviral). The key feature of this discovery was the understanding of the role of those compounds in the sponge. Actually, unlike their analogous found in DNA and RNA, they can be found in a free-state, a fact that led scientists to hypothesize that they were used as a defence mechanism for *Cryptotethia crypta*. The investigations of these compounds led, ultimately, to the antimetabolite concept in pharmacology (molecules structurally similar to the human metabolites that inhibit the use of a metabolite) [69].

Cytarabine was chemically synthesized in 1959 and later produced by fermentation of *Streptomyces griseus*. Curiously, the natural analogue of cytarabine was later isolated from the gorgonian *Eunicella cavolini* [70] but obviously the extraction yield of 0.04% has turned this source economically impractible. Cytarabine continues to be the election drug for the treatment of myeloid leukaemia, non-Hodgkin’s lymphoma and meningeal leukaemia. It may be used alone or in combination with other anticancer agents. Forty years after its entry into the market there is still no better approach to treat these diseases, despite the research efforts to improve therapeutics [69]. However, cytarabine has a short plasma half-life, low stability and limited bioavailability [71], reasons why therapeutic regimens consist in continuous intravenous infusion for seven days, the three first ones in combination with other drugs. This overdosing of patients may lead to side effects, and thus, various prodrug strategies and delivery systems have been exploited extensively to enhance the performance of cytarabine. The first step to improve the bioavailability and stability of cytarabine was the understanding of its mechanism of action and metabolism. Cytarabine has low rates of passive diffusion across membranes and enters into the cells acting as mimetic substrates (antimetabolic agents) for specialized nucleoside transporter proteins. Although the mechanism of action is not completely understood, it appears that cytarabine is converted intracellularly by deoxycytidine kinase, to active cytarabine triphosphate. Activity occurs as the result of inhibition of DNA polymerase via competition with deoxycytidine triphosphate, resulting in the inhibition of DNA synthesis. A limited, but significant, incorporation of cytarabine into both DNA and RNA has also been reported and may contribute to cytotoxic effects [71].

**Table 1:** The marine pharmaceutical clinical pipeline: 2004–2013 evolution.

Compound Name (Trademark)	NP or Derivative	Original NP/Source Organism	Company/Institution (City, State, Country)	Therapeutic Area	Status 2004 [41]	Status 2009 [42]	Status 2013 [67]
Cytarabine (Cytosar-U^®^; Depocyt^®^)	NP derivative	Spongothymidine/sponge *Cryptotethya crypta*	Bedford (Bedford, OH, USA); Enzon (Piscataway, NJ, USA)	Cancer	FDA/EMEA approved	Approved	Approved
Vidarabine (Vira-A^®^)	NP derivative	Spongouridine/sponge *Cryptotethya crypta*	King Pharma (Tenafly, NJ, USA)	Anti-viral	FDA/EMEA approved	Approved	US discontinued
Ziconotide (Prial^®^)	NP	ω-Conotoxin/marine snail *Conus magus*	Elan Corporation (Dublin, Ireland)	Neuropahtic Pain	FDA approved	FDA/EMEA approved	Approved
Omega-3-acid ethyl esters (Lovaza^®^)	NP derivative	Omega-3-fatty acids/fish	GlaxoSmithKline (Brentford, UK)	Hypertriglyceridemia	FDA approved	FDA/EMEA approved	Approved
Trabectedin (Yondelis^®^)	NP	Ecteinascidin 743/tunicate *Ecteinascidia turbinata*	PharmaMar (Colmenar Viejo, Madrid, Spain)	Cancer	Phase II/III	EMEA approved	EMEA approved
Eribulin mesylate (Halaven^®^)	NP derivative	Halichondrin B/sponge *Halichodria okadai*	Eisai (Tokyo, Japan)	Cancer	Phase I	Phase III	FDA/EMEA approved
Brentuximab vedotin (SGN-35) (Adcetris^®^)	NP derivative	Dolastatin 10/sea hare *Dolabella auricularia*	Seattle Genetics (Bothell, WA, USA); Takeda GRDC (Osaka, Japan)	Cancer	-	Phase II	FDA/EMEA approved
Iota-carrageenan (Carragelose^®^)	NP	Iota-carrageenan/red Algee *Eucheuma*/*Cnondus*	Marinomed (Vienna, Austria); Boehringer Ingelheim (Ingelheim, Germany)	Antiviral Viral	-	-	Over-the-counter drug (OTC)
Pliditepsin (Aplidin^®^)	NP	Ascidian *Aplidium albicans*	PharmaMar (Colmenar Viejo, Madrid, Spain)	Cancer	Phase II	Phase II	Phase II/III
PM00104 (Zalypsis^®^)	NP derivative	Jorumycin/sea slug *Joruna funebris*	PharmaMar (Colmenar Viejo, Madrid, Spain)	Cancer	-	Phase II	Phase II
DMXBA (GTS-21)	NP derivative	Anabeseine/worm *Paranemertes peregrina*	Comentis (San Francisco, CA, USA)	Alzhemier’s	Phase I	Phase II	Phase II
Lurbinectedin (PM01183)	NP derivative	Ecteinascidins/tunicate *Ecteinascidia turbinata*	PharmaMar (Colmenar Viejo, Madrid, Spain)	Cancer	-	-	Phase II
CDX-011	NP derivative	Dolastatin 10/sea hare *Dolabella auricularia*	Seatle Genetics (Bothell, WA, USA)	Cancer	-	Phase II	Phase II
SGN-75	NP derivative	Dolastatin 10/sea hare *Dolabella auricularia*	Seatle Genetics (Bothell, WA, USA)	Cancer	-	Phase I	Phase I
PM060184	NP	Sponge *Lithoplocamia lithistoides*	PharmaMar (Colmenar Viejo, Madrid, Spain)	Cancer	-	-	Phase I
Marizomib	NP	Salinosporamide A/Marine actinomycete *Salinispora tropica*	Nereus Pharmaceutical (San Diego, CA, USA)	Cancer	-	Phase I	Phase I
ASG-5ME	NP derivative	Dolastatin 10/sea hare *Dolabella auricularia*	Astellas (Northbrook, IL, USA)	Cancer	-	-	Phase I
Bryostatin I	NP	Bryozoan *Bugula neritina*	NCI (Bethesda, MD, USA)	Cancer Alzheimer’s	Phase I/II	Phase I/II Phase I	Phase I Phase II
Soblidotin	NP derivative	Dolastatin 10/sea hare *Dolabella auricularia*	Aska Pharmaceuticals (Tokyo, Japan)	Cancer	Phase I	Phase III	Discontinued
Synthadotin	NP derivative	Dolastatin 15/Sea hare *Dolabella auricularia*	Genzyme Coporation (Cambridge, MA, USA)	Cancer	Phase I/II	Phase II	Discontinued
*Pseudopterosins*	NP and derivatives	Pseudopterosins */*Soft coral *Pseudoptergorgia elisabethae*	VimRx Pharmaceuticals * (Irvine, CA, USA)	Wound healing	Phase I	Phase II	Discontinued
Elisidepsin (Irvalec^®^)	NP derivative	Kahalides/ *Sea slug Elysia rufescens*	PharmaMar (Colmenar Viejo, Madrid, Spain)	Cancer	-	Phase II	Discontinued
Plinabulin (NPI-2358)	NP derivative	Halimide (NPI-2350)/marine fungus *Aspergillus sp.*	Nereus Pharmaceutical (San Diego, CA, USA)	Cancer	-	Phase II	Discontinued
Tasidotin (ILX-651)	NP derivative	Dolastatin 15/sea hare *Dolabella auricularia*	Genzyme Corporation (Cambridge, MA, USA)	Cancer	-	Phase II	Discontinued
Hemiasterlin	NP	Sponge *Hemiastrella minor*	Eisai (Tokyo, Japan)	Cancer	-	Phase I	Discontinued
Kahalalide F	NP	Sea slug *Elysia rufescens*.	PharmaMar (Colmenar Viejo, Madrid, Spain); Hawai University (Honolulu, HI., USA)	Cancer	Phase I/II	Discontinued	-
Squalamine	NP	Dogphish shark *Squalus acanthias*	Genaera * (Plymouth Meeting, PA, USA)	Cancer	Phase I/II	Discontinued	-
HTI-286	NP derivative	Hemiasterlin/sponge *Hemiastrella minor*	Wyeth * (Philadelphia, PA, USA)	Cancer	Phase I/II	Discontinued	-
Discodermolide	NP	Sponge *Discodermia dissouta*	Novartis (Basel, Switzerland); Harbor Branch (Fort Pierce, FL, USA)	Cancer	Phase I	Discontinued	-
E7389	NP derivative	Halichondria B/sponge *Halichondria okadai*	Eisai (Tokyo, Japan)	Cancer	Phase I	Discontinued	-
Spisulosine (ES-285)	NP	Marine clam *Spisula polynyma*	PharmaMar (Colmenar Viejo, Madrid, Spain)	Cancer	Phase I	Discontinued	-
KRN-7000	NP derivative	Agelasphins/sponge *Agelas mauritianus*	Vrije Universiteit Medical Center, (Amsterdam, Netherlands)	Cancer	Phase I	Discontinued	-
Æ-941 (Neovastat^®^)	NP mixture	Shark cartilage	Æterna (Québec, Québec, Canada)	Cancer	Phase II/III	Discontinued	-
NVP-LAQ824	NP derivative	Psammaplin A/sponge *Aplysinella rhax*	Dana-Farber Cancer Institute (Boston, MA, USA)	Cancer	Phase I	Discontinued	-
Conotoxin G (CGX-1160)	NP	Marine snail *Conus geographus*	Cognetix (Salt Lake City, UT, USA)	Pain	Phase I	Discontinued	-
IPL-576092 and derivatives	NP derivatives	Contignasterol/Sponge *Petrosia contignata*	Aventis * (Strasbourg, France)	Anti-asthmatic	Phase II	Discontinued	-

NP—Natural Product; * extinct companies or purchased by other companies.

As any other molecules, NP can be further improved, either in terms of efficacy and selectivity for the target or achieving optimal pharmacokinetic and pharmacodynamics properties. In the case of cytarabine, these properties were improved by modification of the delivery system. In patients with meningeal leukaemia the inability of cytarabine to cross the blood-brain barrier and exert its action in the brain is very limited [72]. This weakness was overstepped by the formulation of a slow-release liposomal form of cytarabine (DepoCyte^®^) which enables its gradual release, thus ensuring prolonged cytotoxic drug concentrations in cerebrospinal fluid. DepoCyte^®^ is approved in the US and in Europe.

Trying to synthesize a prodrug capable of crossing the cellular membrane without the help of transport proteins, the Oslo-based Clavis Pharma (now Aqualia) has developed a natural fatty acid derivative of cytarabine, the elacytarabine (CP-4055) which can independently enter into leukaemia cells. However CP-4055 failed to surpass phase III trials [73] and the company suspended all on-going development work alleging that there was no significant difference in overall survival of the patients [74].

Vidarabine is an antiviral drug with activity against herpes viruses, poxviruses, and certain rhabdoviruses, hepadnarviruses, and RNA tumor viruses. However, it is more toxic and less metabolically stable than other current antivirals such as acyclovir and ganciclovir; further, it is poorly soluble with low oral bioavailability [75]. It is now obtained from *Streptomyces antibioticus*. However, vidarabine was discontinued in the USA by June 2001 possibly due to its restricted antiviral therapeutic window [42], though is still in use in EU for ophthalmologic applications.

Crucially, more than 40 years after its approval, cytarabine a drug synthesized after a marine natural product, is still in the forefront of cancer drug treatment. By 2007, the global revenues of cytarabine and vidarabine were estimated at $93 M each [76].

#### 4.1.2. Prialt^®^ by Elan Corporation (Dublin, Ireland)

More than 20 years after the entry of cytarabine into the market, ziconotide (Prialt^®^) was granted FDA and EMEA approval in 2004 and 2005 respectively, for the management of severe chronic pain associated with cancer, AIDS and neuropathies. Ziconotide was synthesized in 1987 [17] after its equivalent ω-conotoxin, a naturally occurring peptide, isolated from the venom of the cone snail *Conus magus* [77]. What initially motivated the investigation of cone venoms was the observation that the stings of these molluscs were known to cause human fatality. Later on, the pioneering discovery that cone snails hunted fish, and that different cone snail venoms have different biologically active components led to the research of this organisms and to the isolation and identification of their metabolites, from which ω-conotoxin revealed to be the most promising [78]. As the animal sources did not provide the quantities required to ensure a sustainable supply for clinical profiling it was necessary to accomplish the chemical synthesis, which was achieved by a solid-phase peptide synthesis [79]. It took more then three decades of extensive clinical research in order to be approved by FDA. Ziconotide was the first intrathecal analgesic drug to be aproved after morphine and is one of the most extensively studied new medications. It acts by a new mechanism of action, which consists in the selective *N*-type voltage-sensitive calcium-channel blockade [80]. The ziconotide-induced blockade in the spinal cord inhibits release of pain-relevant neurotransmitters from central terminals of primary afferent neurons. In 2010 Prialt^®^ sales reached $6.1 M [81]. Prialt developemnt prompted the investigation on other *Conus* peptides, several of them having reached human clinical trials [82]. In order to fully exploit the potential of these compounds as drug leads, under the 7th Framework Program (HEALTH 2011-2015) researcher groups of five countries (France, Belgium, Spain, Portugal, Denmark) were granted an european project, the VENOMICS, with a six million euros budget [83].

#### 4.1.3. Lovaza^®^/Omacor^®^ by GlaxoSmithKline (Brentford, UK)

Lovaza^®^ (former Omacor) is the brand name for an anti-hypertriglyceridemia drug, composed by ethyl esters of several omega-3 fatty acids sourced from fish oils, the major constituents being ethyl esters of eicosapentaenoic acid and docosahexaenoic acid. Omega-3 fatty acids of marine origin are well known safe and effective triglyceride lowering agents, and are therapeutic in combination with diet and/or statins [84]. The drug, approved by FDA and EMEA, is commercialized by GlaxoSmithKline but was developed by Reliant Pharmaceutical, through esterification of the natural fatty acids. However it should be differentiated from nonprescription dietary supplement omega-3 preparations, because its production is regulated in a different manner [85].

The approach to this drug discovery was the statistical observation that certain ethnic populations, such as the native Alaskans, had much lower mortality rates from cardiovascular diseases, a fact that was later correlated with their high polyunsaturated fatty acids diets content [86].

#### 4.1.4. Yondelis^®^ by PharmaMar (Colmenar Viejo, Madrid, Spain)

Trabectedin (ET-743, Yondelis^®^) is a novel marine antineoplastic alkaloid with a unique mechanism of action. Its action is not completely understood, but differs from that of traditional alkylating agents as it appears to bind to the DNA minor groove and interact with proteins of the DNA repair machinery [87].It is commercialized by PharmaMar, and co-developed by Johnson & Johnson Pharmaceutical Research and Development andis approved for use in Europe, Russia and South Korea for the treatment of advanced soft tissue sarcoma. It is also undergoing clinical trials for the treatment of breast, prostate, and paediatric sarcomas [88].

The active substance is a natural product, originally isolated from the Caribbean sea squirt *Ecteinascidia turbinata* with a 0.0001% yield, the reason why the structure of the active compound took 30 years to be elucidate [6]. It takes 1 t of animals to isolate 1 g of trabectedin and about 5 g were believed to be needed for a clinical trial [89]. Of course, before trabectedin clinical trials could begin the sustainable supply had to be addressed. The first attempt to assure the production of sufficient quantities of tunicate biomass to support preclinical and clinical needs of trabectedin was the mariculture and aquaculture of *Ecteinascidia turbinata*. This approach provided enough quantity of trabectedin to carry on the clinical development, but revealed to be impractical for commercialization. The cost of aquaculture and deep freezing facilities for biomass from one side, and the low extraction and isolation yield for the other, turned the process economically unfeasible. So, by 1996, Corey and his group provided a total synthesis of trabectedin, but once again experimental procedures represented a enormous barrier to the industrial manufacture. The problem was finally solved with the development of a semi-synthetic industrial viable route to produce trabectedin [89]. This procedure uses safracin B, an antibiotic obtained by fermentation of *Pseudomonas fluorescens*, followed by synthetic transformation with 20 steps and a reported total yield of 1.14% [89]. Nevertheless, this synthetic scheme added a considerable drop of the time and cost of the manufacture. This achievement is a demonstration that synthetic procedures have now reached a high sophistication level which can be successfully used to synthesize in large quantities analogues of NP. Annual sales provided by PharmaMar rounded in 2012 $88 M [90].

#### 4.1.5. Halaven^®^ by Eisai (Tokyo, Japan)

Eribulin mesylate (Halaven^®^) gained FDA’s approval in 2010 and EMEA’s in 2011, for metastatic breast cancer. Halichondrin B is a macrocyclic polyether isolated from the first time in 1986 from the sponge *Halichondria okadai* and found to be highly cytotoxic in murine leukemia cells. As in the case of ziconotide, the difficulty in collecting sufficient material for developmental studies has prevented further evaluation of its clinical application. The total synthesis was achieved by 1991 [91] and subsequent studies identified the macrocyclic lactone C1–C38 moiety as the pharmacophore [92]. This discovery led the researchers from Eisai Research Institute to synthesize a simplified macrocyclic ketone analogue (Eribulin mesylate, former E7389) which kept the same level of activity [93]. Eribulin mesylate and halichondrin B fights cancer cells by inhibiting tubulin, a protein component of the cytoskeleton which is needed to support the rapid growth of cancer cells and is the target of several other cancer chemotherapies, including taxol [6].

#### 4.1.6. Adcetris^®^ by Seattle Genetics (Bothell, WA, USA)

Brentuximab vedotin 63 (Adcetris™) is the latest marine drug to successfully enter into the market. It was was approved for the treatment of Hodgkin and systemic anaplastic large cell lymphoma. It is based on a fully synthetic analog of dolastatin 10 linked to an anti-CD30 antibody. Dolastatin 10 was isolated in 1972, from the sea hare *Dolabella auricularia* in vanishingly small amounts [94]. It was not until 1987 that the structure of the most potent compound in hare extract, dolastatin 10, could be elucidated: 1 t of mollusk biomass was collected from the wild to isolate just 29 mg of dolastatin 10. Only two years later a convergent total synthesis of dolastatin 10 was achieved, which resolved any resupply issues [79]. Later on it was found that dolastatins are, in fact, produced by the cyanobacteria *Symploca hydnoides* and *Lyngbya majuscula*, which are part of the sea hare’s diet [6]. At the time, dolastatin 10 proved to be one of the most potent antineoplastic substances known, with an effective dose that produces the therapeutic response in 50% of the subjects (ED50) in the picomolar range against a number of cancer cell lines. Despite this remarkable activity *in vitro*, dolastatins *in vivo* activity is not sufficient for direct application as drug substances at dosages where toxic side effects are still tolerable. Phase I and II clinical trials of dolastatin 10 and the water-soluble analog auristatin PE were unsuccessful due to lack of efficacy and existance of side effects. Taking advantge of the progress made in the development of antibody-drug conjugates (ADCs) that can selectively deliver cancer drugs to tumor cell, linking the dolastatin 10’ analog, monomethyl auristatin E, to an antibody that targets CD30 (a cell membrane protein present on the surface of Hodgkin’s lymphoma cells) has resulted in the highly effective and well tolerated agent, brentuximab vendotin [79]. An accelerated FDA approval of this agent for use in Hodgkin’s lymphoma and anaplastic large cell lymphoma was granted in August 2011 and the drug is now marketed as Adcetris by Seattle Genetics. It took nearly 40 years from the initial bioactive extract to the approved drug.

#### 4.1.7. Carragelose^®^ by Marinomed (Vienna, Austria)

Carrageenans are a family of linear sulfated polysaccharides that are extracted from red edible seaweeds, mainly *Rhodophyceae* seaweeds. They are widely used in the food industry, for their gelling, thickening, and stabilizing properties. The three main copolymer are designated as Iota, kappa and lambda, according with the number and location of the sulphate moieties on the hexose moiety [95]. Marinomed Biotechnologie GmbH, an Austrian company developed an innovative antiviral nasal spray, containing Iota-carrageenan (Carragelose^®^), that was proven clinically effective against early symptoms of common cold, and is marketed as an OTC drug. It exerts its action creating a protective physical anti-viral barrier in the nasal cavity.

### 4.2. Cosmeceutical Applications

The primary goal of NP research is, in most cases, to discover, develop and foster commercialization of new bioactives for pharmaceutical industry. However, during the course of investigation, some parallel research pathways may emerge, that can make it faster to market a particular product. One of such routes is the application on personal care products. In fact, the borderline between pharmaceutical and cosmetics is getting thinner, and the customer’s demand for innovative, sustainable and truly efficacious products has brought the cosmetic industry to a whole new molecular and subcellular level of development. Cosmeceuticals are a booming business and revenues for this sector were expected to grow at two digits in the next decades [96]. Many years after Shu Uemura first incorporated deep-sea water into its skin care and make-up products, the cosmetics industry’s interest in marine ingredients looks far from ceasing. In fact, in this sector, the marine trend is thriving in the recent years. An example of this can be observed in a simple Google search for “sea water cosmetics” or “marine ingredients cosmetics” that generates approximately 15,000,000 entries. Selected marine-derived actives have started to appear in new prestige skin care launches, including Elemis (The Steiner Group, London, UK), La Prairie (Beiersdorf, Montreux, Switzerland), Crème de la Mer (Estée Lauder, New York, NY, USA), Blue Therapy (Biotherm, Tours, France), amongst many others. This subsection presents the analysis of these products and their paths to the market introduction.

#### 4.2.1. Abyssine^®^ by Unipex (New York, NY, USA)

One of the most common molecular classes of compounds used in the personal care bioactive ingredients sector is the exopolyssacharides (EPS). Various microorganisms produce EPS, including proteobacteria, cyanobacteria and archaea. Marine ecosystems are rich in EPS-producing bacteria that can be isolated from water column, sediments, animals, *etc*. Bacteria producing polymers with novel structures and innovative properties have been isolated in atypical environments, including extreme environments [97,98]. For example, deep-sea hydrothermal vents are areas of active tectonics with diverse physico-chemical characteristics, prone to the development of microorganisms with chemical defence systems against hard environments. Many bacteria living near hydrothermal vents have several types of associations with other organisms (shrimp, worms or mollusks) and some can actually produce EPS under laboratory conditions. These bacteria are mostly found to be part of the genera *Vibrio*, *Alteromonas* or *Pseudoalteromonas* [99,100,101,102,103,104]. Marine bacterial EPS have various physiological roles: they are involved in responses to environmental stress, in recognition processes and cell-to-cell interactions and also in adherence of biofilms to surfaces [105].

**Figure 2:**
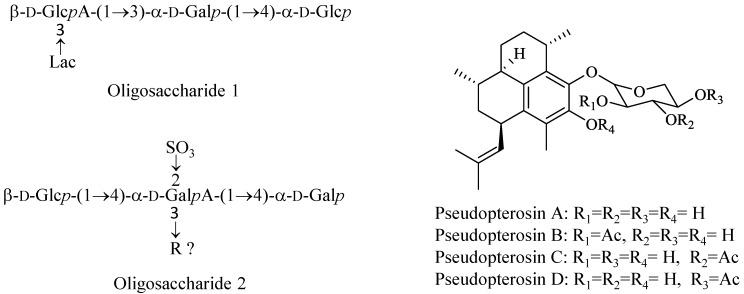
Chemical structures of oligosaccharide 1, oligosaccharide 2 and of pseudopterosins.

Abyssine^®^ by Lucas Meyers is an *Alteromonas* ferment extract containing the EPS HYD657, named deepsane, and it is produced and secreted by the strain *Alteromonas macleodii subsp. fijiensis biovar deepsane* [104]. This strain was collected in 1987, close to a hydrothermal vent located on the East Pacific Rise at 2600 m depth, from a polychaete annelid *Alvinella pompejana* [106]. Although discovered in the late 80’s only in 2012 some light was shed into the chemical structure of deepsane [107], which has been concluded to be a high-molecular-weight polymer of 1.1 × 10^6^ g/mol constituted by two different oligosaccharides (Figure 2).

Several biological screenings were performed in order to evaluate the bioactivity of this particular EPS and it was shown that it effectively protects keratinocytes from inflammatory agents, such as interferon gamma (INF-γ) and intercellular adhesion molecule 1 (ICAM-1). Protective effects have also been demonstrated on Langerhans cells sensitive to ultraviolet attacks and play a major role in the human cutaneous immune defense system [108]. Deepsane has found its market route via applications in cosmeceuticals and is commercially available under the name of Abyssine^®^ (patent PCT 94907582-4) for soothing and reducing irritation of sensitive skin against chemical, mechanical and ultraviolet B (UVB) aggression. This bioactive ingredient is commercialized as a ferment extract and therefore Abyssine^®^ is also rich in minerals, proteins, organic and inorganic compounds, and amino acids.

#### 4.2.2. Resilience^®^ by Estée Lauder (New York, NY, USA)

Resilience^®^ is a line of skin care products from the Estée Lauder company that contains a special extracellular extract from the Caribbean sea whip (gorgonian) *Pseudopterogorgia elisabethae* (Gorgoniidae). This extract is mainly composed by pseudopterosins, which are tricyclic diterpene glycosides (Figure 2). As with many other natural compounds its initial development aimed a pharmaceutical application goal, but it reached the skin care utilization much faster. The pseudopterosins are potent anti-inflammatory and analgesic agents that inhibit eicosanoid biosynthesis by inhibition of both phospholipase A2 (PLA2) and 5-lipoxygenase. Intriguingly, these compounds are found to inhibit only human neutrophils-PLA2, but not PLA2 from other sources [109]. The pseudopterosins have been originally licensed to a SME pharmaceutical company, OsteoArthritis Sciences Inc. (Cambridge, MA, USA), for medical use as potential anti-inflammatory drugs. Preclinical tests for one of the pseudopterosins, a potent topical anti-inflammatory compound, were undertaken and an Investigational New Drug (IND) application filed with the FDA. Additionally, a derivative of a natural pseudopterosin, methopterosin, has completed Phase I and II clinical trials as a wound healing agent. It was, however discontinued, due to the lack of strong effect and drugability. The high lipophilicity and lack of aqueous solubility has hindered the study of the pseudopterosins as drugs for many years. The amphiphilic structure of pseudopterosins would be assumed to impart some aqueous solubility but in fact, the pseudopterosins have little to none and this limits the efficacy of the drug in biological model systems. Several methods have been explored to alter their physical properties (e.g., their solubility) and bioavailability in biological systems including the synthesis of pseudopterosin succinate salts and the production of alternative formulations [6,110].

However, the pseudopterosins original extract has found its earlier way to the marketplace via the personal care route as the regulatory frame allows the use of complete extracts once the efficacy and safety of it as a whole is demonstrated. It was introduced as an additive to prevent irritation caused by exposure to the sun or to chemicals in the Resilience^®^ Estée Lauder cosmetic skin care product [111].

#### 4.2.3. SeaCode^®^ by Lipotec (Barcelona, Spain)

Another example of an EPS with tremendous market impact is the recently launched SeaCode^®^ by LIPOTEC, a mixture of extracellular glycoproteins (GPs) and other glucidic exopolymers produced by biotechnological fermentation of a *Pseudoalteromonas* sp. isolated in the intertidal coasts of Antartic waters [112]. GPs are essential molecules in any living organism, appearing in nearly all biological processes. They consist of polypeptides covalently bounded to oligosaccharide chains (glycans), where the carbohydrate may represent from 1% to 80% of the total mass and can be either *O*-linked or *N*-linked. Consistent with this, GPs possess a large diversity of properties and functions, playing a key role in cellular protein maintenance, cell-to-cell communication, stress recovery and as constituents of cell walls and therefore much sought after for skin care and reconstitution dermal and epidermal purposes. These macromolecules are often significant integral membrane proteins, where they influence cellular interaction. Concretely, GPs mediate the adhesion between cells, which is essential for the development of functional tissues, as well as cell-substrate unions where they act as receptors for adhesion ligands, as it occurs with fibroblasts and fibronectin. This capacity has structural effects when binding cells with proteins like collagen for example, as it offers strength and support to the matrix [113]. Moreover, GPs can act as vehicles (for vitamins and hormones for example), as key hormones (erythropoietin), as enzymes (transferases, oxidoreductases and hydrolases), as protecting and lubricating agents (mucins or elements secreted by the lachrymal and sweat glands), as cryoprotecting molecules that guard from freezing by modifying or avoiding crystals formation, and as vital elements of the immune system (surface compounds of B or T cells with bacteria-binding properties or immunoglobulins) [114]. The combination of GPs and EPS has therefore the potential to provide not also complete protection of the skin but reconstruction effects as well.

To the date, there is no available data on the structural composition of SeaCode^®^. However, in the company page it is stated that SeaCode^®^ is a bioactive ingredient containing ECPs isolated from *Pseudoateromonas* bacteria that proved to ameliorate skin properties by highly enhancing the *in vitro* synthesis of essential dermal proteins (like type I collagen) in Human Dermal Fibroblast, adult (HDFa) cells, maintaining cutaneous tonicity and firmness of those fibroblasts. *In vivo* tests on a panel of volunteers has demonstrated that SeaCode^®^ offers a statistically significant effect in improving skin roughness by 16.5% and 25.1% after 1 and 4 weeks, respectively, mostly at the upper lip wrinkles, due to its replenishing effect.

Besides SeaCode^®^, LIPOTEC has developed other interesting and potent marine derived cosmetic ingredients as Hyadisine®, Antarticine^®^ or even Hyafini^®^, mostly isolated from microorganisms of libraries constructed from samples originated from extreme condition locations.

#### 4.2.4. RefirMAR^®^ by BIOALVO (Lisbon, Portugal)

RefirMAR^®^ by BIOALVO is one of the few, if not the only, marine microorganism derived personal skin care active ingredient with an intracellular origin.

The Mid-Atlantic Ridge (MAR), which extends from the Arctic Ocean to the African Continent, is mainly constituted by submerged mountains and harbors several hydrothermal fields such as Menez Gwen, Menez Hom, Rainbow, Lucky Strike and Mount Saldanha. These vents are under the Portuguese Exclusive Economic Zone (EEZ) and extended continental shelf and have mostly been exploited for its biodiversity composition and geochemical conditions. The organisms surviving in these extreme environments have developed unique and surprising defense functions and this is the basis of many NP developments for many industries [115]. A new bacterial strain from *Pseudoalteromonas* sp. was isolated from one of these extreme vents and characterized.

RefirMAR^®^ is a natural ingredient derived from an intracellular extract produced by biotechnological fermentation of this new *Pseudoalteromonas* sp. strain isolated at near 2300 m depth from the Rainbow vent. Aqueous extraction of the bacterial biomass followed by freeze drying and powder collection results in a complex mixture of macromolecules, mostly proteins, that together act as very potent muscle contraction inhibitor. This defensive function in nature was adapted to cosmetic applications and this extract is the basis of the RefirMAR^®^ ingredient—a potent hydrating, anti-wrinkle and expression lines attenuator comparable to other injectable and/or synthetic solutions. Tests performed in mice synaptossomes showed that this ingredient displays an activity similar to botulinum toxin A (BoNT/A), inhibiting localized muscle contraction by inhibiting acetylcholine release from the neuron-muscle synapsis. This *in vitro* activity was confirmed by *in vivo* assessment of its anti-aging and hydrating potential. RefirMAR^®^ decreases wrinkle depth up to 23% (average 7%) and increases hydration up to 64% (average 34%) after 28 days of topical application [116]. Moreover, RefirMAR^®^ has the major advantage of being suitable for topical application, with no need to make use of unpleasant injections for getting the desired effect (data not shown). Structural data on RefirMAR^®^ is not yet available.

Despite RefirMAR^®^ obvious pharmaceutical potential application to disorders where neuromodulation and acetylcholine release inhibition can play a role in disease control, BIOALVO has chosen to firstly develop this bioactive for applications in the cosmeceutical market. This choice was grounded on the fact that this is a faster route to market, a detail very important for small companies to be able to survive by using the cash from sales generated by this cosmetic route to finance the much more costly and long paced pharmaceutical development.

#### 4.2.5. Microalgae Derived Bioactive Ingredients

Recently, microalgae conquered their space in the cosmetics arena and are the source of some of the most innovative solutions in our products today. Microalgae are microscopic unicellular organisms capable to convert solar energy to chemical energy via photosynthesis. The potential of microalgae photosynthesis for energetic use is widely recognized due to their more efficient utilization of sunlight energy compared with higher plants [117]. They also produce a wide range of bioactive metabolites such as proteins, lipids, carbohydrates, carotenoids or vitamins that can be exploited for commercial use in food or cosmetic industry. Some microalgae species are established in the skin care market with the main ones being *Arthrospira* and *Chlorella* [21,118]. Microalgae extracts are incorporated in many face and skin care products (e.g., anti-aging cream, refreshing or regenerating care products, emollient and anti-irritant in peelers), sun protection and hair care products. Microalgae derived new polysaccharides, especially if sulfatated, are particularly interesting because they could offer a promising and innovative alternative to the existing seaweed-derived ones, and underlie the development of new functional cosmeceutical products.

Some of the most recent advances in novel microalgae functional bioactives include Dermochlorella DG^®^, XCELL-30^®^, Alguronic Acid^®^ and Alguard^®^.

Dermochlorella DG^®^ from CODIF Reserche & Nature (Britany, France), a *Chlorella* sp. extract containing oligopeptides acts as restructuring actives on the dermal—epidermal junction to increase firmness and skin tone. The active also acts on the epidermis to erase vascular imperfections as it boosts collagen production into blood vessels (dark circles and small vascular imperfections) [119].

XCELL-30^®^ from Greensea (Mèze, France) is developed from microalgae endemic to Madagascar, and specifically acts on cellular turnover in the basal layer of the epidermis, thus allowing the preservation of the youthful characteristics of the skin [120].

Alguronic Acid^®^ from Algenist (San Francisco, CA, USA), a novel microalgae powerful compound is responsible for regenerating and protecting the microalgae cell in harsh environments and conditions. When scientifically tested, Alguronic Acid^®^ demonstrated significant anti-aging properties, helping to rejuvenate the skin for a more youthful appearance [121].

Alguard^®^ is a natural sulfated polysaccharide compound isolated from a single red microalgae (*Porphyridium* sp.*)*, that acts as a shield, creating a thick protective layer around the cell and protecting it in its intertidal extreme environment. Research has revealed a wealth of biological activities, demonstrating that Alguard^®^ is not merely a physical barrier but an active protection against photo damaging, ageing and micro abrasion of the skin [122].

#### 4.2.6. Other Derived Marine Bioactive Ingredients

In addition to the specialty ingredients mentioned above, the marine environment contains a source of polyunsaturated fatty acids (PUFAs), polysaccharides, essential minerals and vitamins used as cosmeceuticals. Potassium alginate and fucoidan from brown algae, aluminum silicate from sea mud, chitin from crustaceans, shell’ powder from oysters and carrageenan from red algae are some examples of less differentiated but widely used marine active ingredients. Among marine organisms, sea algae have been identified as a source of cosmeceuticals due to their high levels of vitamins and minerals. An Italian company called Lacote has in fact a vast range of skin care products using Guam algae. Also the German company OceanBasis possesses a huge portfolio of algae-based cosmeceuticals that derived from its aquaculture farm in the Baltic Sea. The certified farm provides a sustainable source of active ingredients for its Oceanwell line. *Laminaria japonica*, is known to store marine minerals in a highly concentrated form, and has been used to generate special algae-based ingredients active as ultraviolet radiation damage protectors. In addition, this alga is also known for the production of effective moisture binding agents that protect them against dehydration, a feature that may prove to be harnessed in the future Another example is the use of extracts of *Undaria* algae which are known to improve the condition of the extracellular matrix that keep the skin dense and plump while circulating water throughout epidermis. Sea mud contains various nutrients and minerals making it recognized for its therapeutic properties against psoriasis and other skin-related disorders [24]. Another example is marine phytoplankton which is known to contain more than 65 aminoacids, essential fats, vitamins and key minerals, among other active ingredients with cellular regeneration capacities. New trends are pointing towards the use of food sources such as marine fish-derived collagen and gelatine as excellent functional ingredients for the cosmetic industry [123]. In fact the high moisturizing properties of these compounds make them suitable as novel ingredients for cosmetic creams and gels.

Hence, due to the ability to create such interesting ingredients, marine skin care is truly a wave of future in beauty.

## 5. How Can Success Rate Be Improved?

It is unquestionable the need to unlock the potential for innovation contained in the vastly unexploited ocean habitats, but if we could increase the market entry success rates of what is currently being done by just 10%, instead of dozens of unique MNP (or derived thereof) we could have hundreds of hits reaching the market. The focus of this section is then on what can be learned from the successful and unsuccessful stories that can be applied to novel and/or ongoing MNP discovery and development programs.

**Figure 3:**
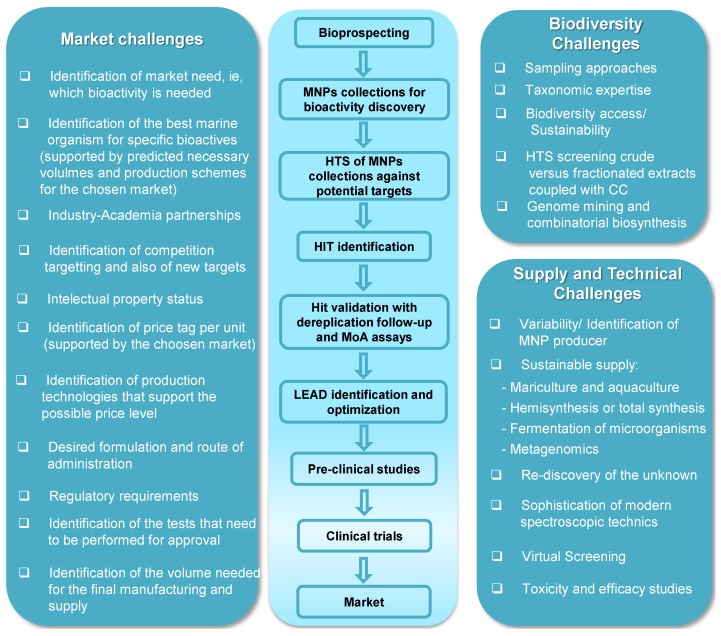
Marine Natural Products: from bioprospecting to market, highlighting biodiversity, supply and technical and market challenges faced during the process.

These success-learning points can be grouped in the same sections of the previously identified challenges—what can be made to improve biodiversity access and make more meaningful and productive screening programs (biodiversity); how can alignment between isolation, mechanisms of action and production schemes with commercial success be assured (supply and technical) and finally, how can cost be contained and success rates of market entry be increased [46]. In Figure 3 it is depicted the process of getting a marine natural product into the market having in consideration these important challenges.

### 5.1. Biodiversity Challenges

The access to the ocean and to the deepest of its spots remains very difficult and it is thus important to focus on optimizing sampling strategies [124]. Further robotic and engineering technology is needed to fully evaluate the oceans’ available biodiversity. More technology is needed allowing the development of more sophisticated submersibles and ROVs that can go deeper in the ocean but, at the same time, that make these types of robotics more accessible to a wider community of users, democratizing its access. With the adequate equipment, collection in locations never reached before will enable the identification of novel organisms and potentiate the findings of novel structures with new market applications. To learn more about the original environment of the organisms and the physic-chemical conditions of their habitat, and to integrate this knowledge directing it to potential inferred activities is, in fact, a current strategy many successful programs have taken to identify potential novel marine bioactives. Efforts are also needed to improve marine taxonomic expertise, employing both classical and molecular methods, to speed up the knowledge on species diversity, gaining competitiveness and making marine biodiscovery more efficient. Harmonization of Nagoya protocol’s application in different countries, with perhaps tighter control supervision by national jurisdictions, and a clear access scheme to this genetic biodiversity by non-locals is needed to boost industry interest and developments in this arena.

However, having access to a wider number of samples and novel organisms does not represent *per se* a guaranty of increasing success rates. The development of strategic and market oriented screening programs is a must for shorter market entry and higher success chances. More meaningful and productive screening programs are the key to finding new hits and leads in natural product libraries that can actually generate a new product. The appropriation of CC and HTS techniques for increasing the efficiency of NP discovery is paramount. CC can be directed to NP leads development when derivatization is the way to develop a novel bioactive with natural inspiration. On the other hand, improving HTS assays and platforms target or mechanism specific and sensitive and robust enough to allow usage of limited quantities, while eliminating poorly performing samples, can truly be the answer to handle low concentration actives and thousands of samples simultaneously. Additionally, the success rates of hit identification can be improved by screening simultaneously pre-fractionated libraries and crude extract libraries [3] and performing an integrated analysis of the results. Pre-fractionation can eliminate compounds too hydrophilic or hydrophobic, less drug-like, and compounds found in each fraction can be tested in detectable concentrations. Also, the reduced complexity can reduce the number of cycles of bioassay-guided fractionation needed to isolate and identify the active components [125]. Pure natural product libraries are considerably more expensive and difficult to obtain, but offer the advantage of a straightforward hit detection and increasing the number of such libraries is also a good strategy for the field.

An alternative approach to generate new molecules or scaffolds from marine origin is combinatorial biosynthesis, in which genes responsible for individual metabolic reactions from different organisms are combined to divert metabolic pathways towards novel products that were previously inaccessible or difficult to obtain [126]. Ultimately, marine organisms themselves might be metabolically engineered. Combinatorial biosynthesis can be achieved by biotransformation, in which the compounds are modified chemically by biocatalysts, mutasynthesis or feeding of compounds to mutant biocatalysts, combinatorial metabolism in hybrids, activation of silent metabolism and combinatorial piling up of genes in heterologous hosts. To exploit the full potential of this biotechnological approach, it is essential the knowledge of the genes involved in the biosynthesis, that is to say a comprehensive understanding of secondary metabolic pathways [127]. Unfortunately, these pathways are far from being understood. In the case of marine organisms, apart from the common difficulties associated to biosynthetic studies, the role of microbial contamination or symbionts in the biosynthesis of some metabolites must be clarified. The future of combinatorial biosynthesis, which can play an important role improving both diversification and chemical and natural product libraries, lies on elucidation of metabolic pathways, engineering efforts to create more robust expression systems and genetic tools [126]. Finally, a more democratic access to complete natural compounds databases could improve the developments of novel programs.

Biodiversity access is important to unlock the total potential of the marine environment to solve today’s current problems and needs, and serious work has to be done by governmental politics, academic and industry researchers in order to improve bioprospecting activities. Improving the success and meaning of screening programs and directing the technologies to better applications while focusing on clear market strategies is a must for increasing success chances of each bioactive hit identified.

### 5.2. Supply and Technical Challenges

If to access the total available marine biodiversity is a challenge that is only addressable with investment and further technology development, to ensure a continuous flow of material and reproducible bioactive manufacturing is a completely different issue. This issue should be given serious consideration at the beginning of any natural product discovery program, as choosing between micro or macroorganisms can alter dramatically the elected development strategy.

From the analysis of Table 1 one can pick a striking feature between the role of marine drugs in the former years and the one it has now. As it was stressed, for the first marine drugs (cytarabine, vidarabine, ziconotide, trabectedin, eribulin mesylate and brentuximab vedotin) a gap of 20 to 40 years passed between the discovery of the active molecule and commercialization of the drug. The same happened with the use of marine bioactive ingredients usage in cosmeceuticals, as their botanical counterparts’ access and supply was, for decades, much easier. The supply issue was, with any doubt, the main shortcoming that development of these bioactives has faced, that was only overcome when synthetic procedures or biotechnological production reached a high level of progress. The structural complexity of NP, of which chirality is one of the most important aspects, posed significant synthetic challenges that slowed the drug discovery process. However, if one looks to the compounds that entered in clinical trials or in the cosmeceutical markets in the past decade, the time elapsed between the discovery of the molecule (or of its activity) and the sustainable production was greatly improved. Creative approaches to by-pass the lack of sufficient bioactive natural compound have been undertaken, and today there is no reason for supply still being a liability of MNP as leads for new bioactives.

One other major route used to surpass the limitation of raw biological material needed for a successful product development is the increased focus on marine microorganisms, since fermentation and scale up of production processes using cultured microorganisms can eliminate this supply problem. The challenge of accessing the total microorganisms’ biodiversity and achieving to culture all of them in the lab is still there, but recently production of many until now unculturable microorganisms was successfully achieved, via novel culturing methods or metagenomics approaches. Nevertheless, further improvements still have to be made regarding the identification, cloning, genetic manipulation and expression of biosynthetic pathways in order to apply these methods for production of identified NP. In addition, metagenomics tecnhiques can also be applided so that NP can be identified and used as leads even when deriving from unculturable microorganisms. At the actual state of the art, choosing microorganisms is therefore a good strategy for a faster commercial success.

In the case of macroorganisms, synthetic or hemisynthetic approaches are almost always undertaken, and so it is advisable to integrate early on the development strategy the concept that a commercial viable synthesis must be as short as possible, a balance must be achieved between the yield and the steps needed to complete the process, and intermediate purification steps must be avoided. Sophistication of modern spectroscopic technics, mainly NMR, enables the identification of molecules on the nanomolar and even smaller scale, allowing the exploration of marine species existing in very low biomass in nature, such as thinly encrusting invertebrates or microalgae slimes [6]. Nevertheless, it is very important to improve structure elucidation using the most rigorous and unequivocally methods available; cross-checking of the structure in light of current biosynthetic knowledge and understanding is also advisable [58]. Additionally, choosing species that are less susceptible to environmental influence or prone to lab reproduction under more controlled conditions is a plus. For example, recent developments in reproduction cloning of corals in aquaculture are a good illustration of a strategy to overcome macroorganisms limitations when it comes to consistent raw material supply [128].

The recent progresses in molecular biology and assay developments have also shed light into MNP potential efficacy and modes of action. Integrating secondary more complex systems into the screening processes has helped preventing progression of toxic or non-stable substances through the development pipeline and is nowadays a common practice. This is particularly true in the current MNP drug pipeline [67]. As it was pointed out earlier, the major causes of discontinuation of compounds from clinical trials are lack of efficacy and drug toxicity. Hence, a major focus on mode of action (MoA) determination and incorporation of solid secondary screening early on the discovery programs is demanded for success.

### 5.3. Market Challenges

If the previous described categories of challenges are successfully managed, the chances of reaching the market with a viable marine bioactive increase substantially. Parallel to the choice of the desired type of microorganism and technical and supply aspects needed to ensure an efficacious and sustainable bioactive, market guidelines must be incorporated into the program. Several very important questions must be integrated from day one in marine drug discovery programs: (i) what market needs will be addressed by the discovery program, what is the competition segment and if is there space for novel targets; (ii) is it possible to produce novel functional bioactives, can delivery innovation be achieved and how is the intellectual property in that field; (iii) what is the price tag per kilogram aimed at, or supported by the chosen market; (iv) which production technologies will support the target price; (v) how much volume is needed for the final manufacturing and supply in accordance to those market goals; and finally (vi) what are the regulatory requirements and tests needed to successfully approve the bioactive.

Being the academic focus and the industry goals sometimes too apart, greater efforts must be made to bring these two worlds together. Some of the recent successes in marine natural product developments have only been made possible due to close collaborations between academic and industrial partners. Results of this strategy are now coming to market (e.g., Yondelis^®^ and RefirMAR^®^) being an example of the synergistic effect of these alliances. The European 7th Framework Programme and other SME/academia incentive schemes have greatly contributed to this collaborative research. The next European program—Horizon 2020 will reinforce this trend through several incentives to academia/SMEs and industry collaborations. The best solution to develop successful marine bioactives is to design, from the beginning, joint academic-industry discovery and development programs. This approach combines the expertise of excellent academic groups with deep knowledge of marine life, analytical and synthetic technics, *etc*., with the industry and SME’s fast development needs, market awareness and business expertise. Only with this integrated approach, this science cluster can be pushed forward and contribute to building innovative solutions for today’s societal needs, using the ocean as the next frontier of development.

## 6. Conclusions

The marine environment represents a relatively untapped source of biologically active compounds that can be applied in pharmaceutical and cosmeceutical industries, among others. The recent advances in natural products expertise, underwater exploration and bioassays, as well as the improvement of the technology available for isolation and cultivation of marine microorganisms, have enormously contributed to enkindle the interest in exploiting the biodiversity locked in the ocean.

However MNP discovery and development programs still face several challenges connected with biodiversity access, sustainable supply, technical support and market access, which have been outlined in this paper. When starting a new endeavor to the sea, careful consideration must be given to those challenges, especially if the goal is to commercialize a compound. A market orientation and an industrial/upscale mindset is one of the keys to improve success rates in this field. To identify the specific bottlenecks associated with each program and to incorporate feasible alternatives is a strategy that can lead successfully to unlock novel “ocean solutions” to today’s societal challenges. Marine resources with all its facets comprise, undoubtedly, a huge economic potential for the world and represent a sector which can deliver ‘smart, sustainable and inclusive growth’, a core objective of the Europe 2020 Strategy.
